# Supplementary material for: Identification of photoperiod-induced specific miRNAs in the adrenal glands of Sunite sheep (Ovis aries)
Source: Front Vet Sci. 2022 Jul 22;9:888207. doi: 10.3389/fvets.2022.888207 (PMC9354845; doi:10.3389/fvets.2022.888207)
Supplement: Supplementary file 1 [file Data_Sheet_1.ZIP › Supplementary file/miRDeep2 file.zip!/miRDeep2 file/Novel_Structure/SP42A3/Novel_328.pdf]

| | | |
|---|---|---|
| Provisional ID | : | NC_019460.2_7246 |
| Score total | : | 20.1 |
| Score for star read(s) | : | 3.9 |
| Score for read counts | : | 12.4 |
| Score for mfe | : | 2.3 |
| Score for randfold | : | 1.6 |
| Score for cons. seed | : | |
| Total read count | : | 36 |
| Mature read count | : | 17 |
| Loop read count | : | 0 |
| Star read count | : | 19 |

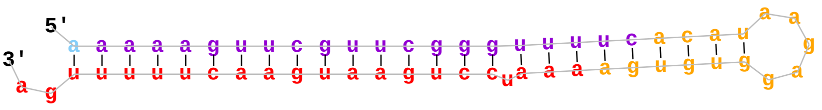


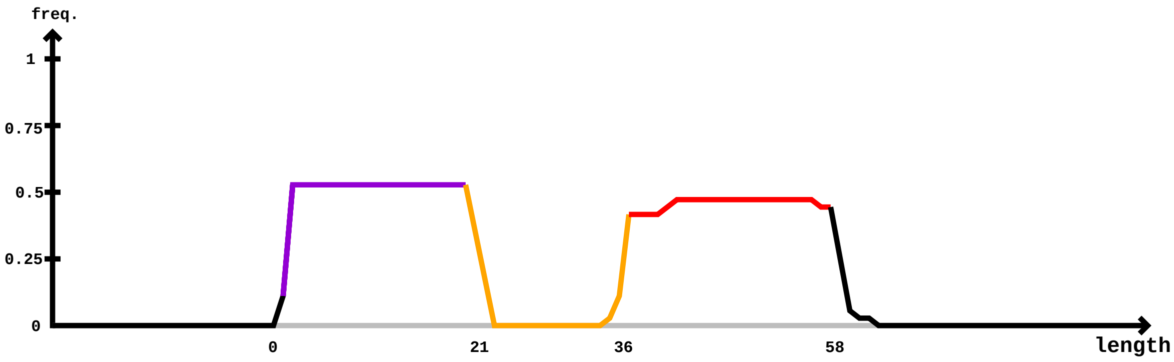


```
                                           Star                              Mature
5'- aguacuuuguauuggguuggugaaaaaguucguucggguuuucacauaagaggugugaaaauccugaaugaacuuuuugaccaacccaaucuuugucuuuccccucuacuag   -3'    obs
    aguacuuuguauuggguuggugaaaaaguucguucggguuuucacauaagaggugugaaaauccugaaugaacuuuuugaccaacccaaucuuugucuuuccccucuacuag          exp
    ((.((...(.(((((((((((.(((((((((((((((((((((((((.....))))))))).)))))))))))))))).))))))))))))...))))..............    reads      mm          sample
    ......................aaaaaguucguuUggguuuuc.....................................................................    3          1           seq
    ......................aaaaaguucguucggguuuuc.....................................................................    1          0           seq
    .......................aaaaguucguucggguuuu......................................................................    7          0           seq
    .......................aaaaguucguucggguuuuc.....................................................................    8          0           seq
    ........................................................gaaaaCccugaaugaacuuuuu..................................    1          1           seq
    .........................................................aaaaAccugaaugaacuuuuuga................................    3          1           seq
    ..........................................................aaaAccugaaugaacuuuuuga................................    11         1           seq
    ..............................................................ccugaaugaacuuuuugacU..............................    1          1           seq
    ...............................................................cugaaugaacuuuuugaccaC............................    1          1           seq
```
